# Worse prognosis of *KRAS *c.35 G > A mutant metastatic colorectal cancer (MCRC) patients treated with intensive triplet chemotherapy plus bevacizumab (FIr-B/FOx)

**DOI:** 10.1186/1741-7015-11-59

**Published:** 2013-03-04

**Authors:** Gemma Bruera, Katia Cannita, Daniela Di Giacomo, Aude Lamy, Thierry Frébourg, Jean Christophe Sabourin, Mario Tosi, Edoardo Alesse, Corrado Ficorella, Enrico Ricevuto

**Affiliations:** 1 Medical Oncology, S. Salvatore Hospital, University of L'Aquila, Via Vetoio, L'Aquila, 67100, Italy; 2 Department of Biotechnological and Applied Clinical Sciences, University of L'Aquila, Via Vetoio, L'Aquila, 67100, Italy; 3 Laboratory of Tumor Genetics, University Hospital, 1 rue de Germont, 76031 Rouen, France; 4 INSERM U614, University of Rouen, 22 Boulevard Gambetta, 76183 Rouen, France; 5 Department of Pathology, INSERM U614, Rouen University Hospital, 1 rue de Germont, 76031 Rouen, France

**Keywords:** *KRAS *mutation, *Kras *c.35 G > A mutation, triplet chemotherapy plus bevacizumab, metastatic colorectal cancer, FIr-B/FOx

## Abstract

**Background:**

Prognosis of *KRAS *wild-type and mutant metastatic colorectal cancer (MCRC) patients (pts) treated with bevacizumab (BEV)-containing chemotherapy is not significantly different. Since specific *KRAS *mutations confer different aggressive behaviors, the prognostic role of prevalent *KRAS *mutations was retrospectively evaluated in MCRC pts treated with first line FIr-B/FOx, associating BEV to triplet chemotherapy.

**Methods:**

Tumor samples were screened for *KRAS *codon 12, 13 and *BRAF *V600E mutations by SNaPshot and/or direct sequencing. MCRC pts <75-years-old were consecutively treated with FIr-B/FOx: weekly 12 hour-timed-flat-infusion/5-fluorouracil (900 mg/m^2 ^on days 1,2, 8, 9, 15, 16,22, 23), irinotecan plus BEV (160 mg/m^2 ^and 5 mg/kg, respectively, on days 1,15); and oxaliplatin (80 mg/m^2^, on days 8,22). Pts were classified as liver-limited (L-L) and other/multiple metastatic (O/MM). Progression-free survival (PFS) and overall survival (OS) were compared using the log-rank test.

**Results:**

Fifty-nine pts were evaluated at a median follow-up of 21.5 months. *KRAS *mutant pts: c.35 G > A, 15 (25.4%); c.35 G > T, 7 (11.8%); c.38 G > A, 3 (5%); other, 3 (5%). *KRAS *wild-type, 31 pts (52.7%). The objective response rate (ORR), PFS and OS were, respectively: c.35 G > A mutant, 71%, 9 months, 14 months; other than c.35 G > A mutants, 61%, 12 months, 39 months. OS was significantly worse in c.35 G > A pts compared to *KRAS *wild-type (*P *= 0.002), *KRAS/BRAF *wild-type (*P *= 0.03), other MCRC patients (*P *= 0.002), other than c.35 G > A (*P *= 0.05), other codon 12 (*P *= 0.03) mutant pts. OS was not significantly different compared to c.35 G > T *KRAS *mutant (*P *= 0.142).

**Conclusions:**

*KRAS *c.35 G > A mutant status may be significantly associated with a worse prognosis of MCRC pts treated with first line FIr-B/FOx intensive regimen compared to *KRAS/BRAF *wild type and other than c.35 G > A mutant pts.

## Background

*KRAS *genotype, wild-type or mutant, addresses the medical treatment of metastatic colorectal cancer (MCRC) patients (pts), consisting of triplet regimens combining chemotherapeutic drugs, or doublets plus targeted agents [1]. The addition of anti-epidermal growth factor receptor (anti-EGFR) treatment is not effective in *KRAS *mutant patients [2,3]; anti-vascular endothelial growth factor (anti-VEGF) treatment added to doublet chemotherapy was effective in *KRAS *wild-type and mutant pts [4,5]. In liver limited (L-L) MCRC, these first line options, integrated with secondary resection of liver metastases, may significantly increase survival [6-13].

The prognostic relevance of the *KRAS *genotype can be assessed by evaluation of clinical outcome (progression-free survival (PFS), overall survival (OS)) in wild-type and mutant pts, depending on differential tumor biological aggressiveness and predictive effectiveness of treatment strategies. The median OS of *KRAS *wild-type and mutant MCRC pts treated with irinotecan, 5-fluorouracil and leucovorin (IFL) plus bevacizumab (BEV) was 27.7 and 19.9 months, respectively [4,5]. The hazard ratio (HR) for risk of death was 0.51 and statistically significant only when *KRAS *and *BRAF *wild-type pts were compared with pts harboring mutations in one gene. In *KRAS *wild-type pts and in *BRAF *wild-type pts compared to mutant, HR was 0.64 and 0.38, respectively, but did not reach statistical significance [4]. Recently, phase II studies proposed by Masi *et al*. [8], and by our group [7], showed that intensive medical treatment consisting of triplet chemotherapy plus BEV, according to FOLFOXIRI plus BEV and FIr-B/FOx schedules, respectively, may increase the activity and efficacy of the treatment in MCRC pts with the *KRAS *wild-type and mutant genotypes [8,13]. Median OS of pts treated with FIr-B/FOx was different in *KRAS *wild-type and mutant pts (38 months and 21 months, respectively), but not significantly [13]. L-L pts compared to other/multiple metastatic (O/MM) pts achieve significantly increased PFS and OS; in addition, *KRAS *wild-type pts with L-L disease may achieve a significantly greater benefit from integration with liver metastasectomies, with respect to *KRAS *mutant patients [11,13].

The *KRAS *wild-type genotype predicts favorable clinical outcomes when anti-EGFR or anti-VEGF molecules are added to doublet chemotherapy [2,5]. The *KRAS *mutant genotype significantly predicts prolonged PFS up to 9.3 months, while there was no increase in OS and activity [14,5], in pts treated with BEV added to IFL compared to IFL.

*KRAS *mutations occur in 35% to 45% of colorectal cancer (CRC), mostly in codon 12 (80%), c.35 G > A (G12D) and c.35 G > T (G12V) transversions, representing 32.5% [14,15] and 22.5% [14,16], respectively, and codon 13, predominantly c.38 G > A (G13D) mutations [17]. These mutations impair the intrinsic GTPase activity of KRAS, thus leading to constitutive, growth-factor-receptor independent activation of downstream signalling [18]. In the *in vitro *model proposed by Guerrero *et al*. [19], codon 12 mutations increase aggressiveness by the differential regulation of KRAS downstream pathways that lead to inhibition of apoptosis, enhanced loss of contact inhibition and increased predisposition to anchorage-independent growth [19]. Codon 13 mutations showed reduced transforming capacity compared to codon 12 mutations [20].

The biological aggressiveness of codon 12 *KRAS *mutant tumors seems to confer worse clinical behavior. A multivariate analysis suggested that the presence of *KRAS *mutation significantly increased the risk of recurrence and death; the codon 12 c.35 G > T (G12V) mutation retained an independent increased risk of recurrence and death [21], and significantly reduced disease-free survival and OS of Dukes C pts [16]. The poorer prognosis conferred by codon 12 *KRAS *mutations was not confirmed in other studies [22,23].

We report a retrospective exploratory analysis evaluating the prognostic value of the prevalent codon 12 c.35 G > A (G12D) *KRAS *mutation in MCRC pts enrolled in a previously reported phase II study [7] and in an expanded clinical program proposing FIr-B/FOx intensive regimen as first line treatment.

## Methods

### Patient eligibility

MCRC pts were enrolled in a previously reported phase II study [7] and in the expanded clinical program proposing FIr-B/FOx association as first line treatment. The study was approved by the Local Ethical Committee (Comitato Etico, Azienda Sanitaria Locale n.4 L'Aquila, Regione Abruzzo, Italia) and conducted in accordance with the Declaration of Helsinki. All patients provided written, informed consent.

### Schedule

FIr-B/FOx association consisted of 5-fluorouracil associated with alternating irinotecan/BEV or oxaliplatin, according to a previously reported weekly schedule [7].

### Mutation analysis

*KRAS *and *BRAF *analyses were performed on paraffin-embedded tissue blocks from the primary tumor and/or metastatic site. Genotype status was analyzed for *KRAS *codon 12 and 13 mutations and *BRAF *c.1799 T > A (V600E) mutation by SNaPshot^® ^multiplex [13,24], for *KRAS *mutations and *KRAS/BRAF *mutations in 36 and 32 samples, respectively; direct sequencing of the *KRAS *gene was performed in 23 samples. After treatment with xylene thiocyanate and selection of tumor cell clusters, DNA was isolated using the RecoverAll™ Total Nucleic Acid Isolation Kit for FFPE Tissues (Applied Biosystems, Courtaboeuf, France) according to the manufacturer's instructions.

### SNaPshot^® ^assay

SNaPshot^® ^multiplex assay was performed as previously reported [13,25]. *KRAS *exon 2 and *BRAF *exon 15 were simultaneously PCR-amplified and analyzed for the presence of mutations at *KRAS *nucleotides c.34G, c.35G, c.37G, c.38G and *BRAF *mutation at nucleotide c.1799T using the ABI PRISM SNaPshot^® ^Multiplex kit (Applied Biosystems, Foster City, CA, USA) [13,25]. Labelled products were separated on 36 cm-long capillaries in POP7 polymer during a 25-minute run in an ABI Prism 3130*xl *Genetic Analyzer (Applied Biosystems). Data were analyzed using the GeneMapper Analysis Software version 4.0 (Applied Biosystems).

### Direct sequencing assay

*KRAS *exon 2 sequence reaction was performed from PCR-amplified tumor DNA, using the Big Dye V3.1 Terminator Kit (Applied Biosystems), and run on an automated sequencer (ABI 3130, Applied Biosystems).

### Study design

A retrospective analysis has been planned to evaluate the prognostic relevance of the prevalent codon 12 c.35 G > A (G12D) *KRAS *mutant genotype on the clinical outcome of MCRC pts treated with first line FIr-B/FOx. Pts were classified as L-L and O/MM [13]. Clinical criteria of activity and efficacy were ORR, PFS and OS. ORR was evaluated according to Response Evaluation Criteria in Solid Tumors (RECIST) criteria [26]; pathological complete response was defined as absence of residual cancer cells in surgically resected specimens. Overall activity of integrated medical treatment and secondary liver surgery, consisting of the sum of clinical complete responses (cCR) and liver metastasectomies was also evaluated, as previously reported [11]. PFS and OS were evaluated using the Kaplan and Meier method [27]. The log-rank test was used to compare PFS and OS in different subgroups of pts [28]. PFS was defined as the length of time between the beginning of treatment and disease progression or death (resulting from any cause) or to last contact; OS, as the length of time between the beginning of treatment and death or to last contact. Clinical evaluation of response was made by computerized tomography (CT)-scan; positron emission tomography (PET) was added based on the investigators' assessment. Pts with L-L metastases were evaluated at baseline and every three cycles of treatment, by a multidisciplinary team (medical oncologist, liver surgeon, radiologist) to dynamically evaluate resectability, defined according to previously reported resectability categories [11]. The resection rate was evaluated in intent-to-treat population enrolled. Liver metastasectomies were defined as: R0, if radical surgery; R1, if radiofrequency was added.

## Results

### Patient demographics

*The KRAS*/*BRAF *genotype was evaluated in 59 pts, among 64 consecutive, unselected MCRC pts recruited in the phase II study and expanded clinical enrollment of the FIr-B/FOx regimen as the first line treatment of MCRC [7,13]: 31 pts (53%) were identified as *KRAS *wild-type and 28 (47%) as *KRAS *mutant [13]. The prevalence of *KRAS *mutations was: codon 12, 24 pts (40.6%), specifically c.35 G > A (G12D), 15 pts (25.4%), c.35 G > T (G12V), 7 pts (11.8%), c.34 G > A (G12S) and c.35 G > C (G12A), 1 patient each; codon 13, 4 pts (6.7%), c.38 G > A (G13D), 3 pts (5%) and c.37_39 dupl, 1 patient (Table 1).

**Table 1:** *KRAS *mutations

*KRAS *mutant					
**Exon**	**Codon**	**Hot spot site**	**Amino acid**	**No. of patients**	**%**

2	12			24	40.6
		c.34 G > A	p.Gly12Ser	1	1.6
		c.35 G > A	p.Gly12Asp	15	25.4
		c.35 G > T	p.Gly12Val	7	11.8
		c.35 G > C	p.Gly12Ala	1	1.6
	13			4	6.7
		c.37_39 dupl	p.Gly13dupl	1	1.6
		c.37	-	-	-
		c.38 G > A	p.Gly13Asp	3	5

Table 2 describes the demographic and baseline features of pts with the c.35 G > A *KRAS *mutation, other *KRAS *mutations, and *KRAS *wild-type: male/female ratio, 11/4, 5/8 and 21/10, respectively; liver metastases, 12 (80%), 8 (61.5%) and 19 pts (61%), respectively.

**Table 2:** Patients' features

	c.35 G>A *KRAS *mutant	Other *KRAS *mutant	*KRAS *wild-type
	
	Total Number (%)	Total Number (%)	Total Number (%)
Number of patients	15 (25)	13 (22)	31 (53)
Sex			
male/female	11/4	5/8	21/10
Age, years			
median	67	63	64
range	51 to 73	48 to 71	42 to 73
≥65 years	8 (53)	5 (38)	13 (42)
WHO Performance Status			
0	13 (87)	13 (100)	28 (90)
1-2	2 (13)	-	3 (10)
Metastatic disease			
metachronous	5 (33)	2 (15)	10 (32)
synchronous	10 (67)	11 (85)	21 (68)
Primary tumor			
colon	10 (67)	10 (77)	14 (45)
rectum	5 (33)	3 (23)	17 (55)
Sites of metastases			
liver	12 (80)	8 (61.5)	19 (61)
lung	3 (20)	2 (15)	7 (23)
lymph nodes	4 (27)	4 (31)	10 (32)
local	2 (13)	1 (8)	6 (19)
other	4 (27)	2 (15)	2 (6)
Number of involved sites			
1	8 (53)	9 (69)	17 (55)
≥2	7 (47)	4 (31)	14 (45)
Single metastatic sites			
liver-limited	6 (40)	7 (54)	12 (39)
other than liver	2 (13)	2 (15)	7 (22)
lung	1 (6.5)	1 (8)	2 (6)
lymph nodes	-	1 (8)	2 (6)
Local	1 (6.5)	-	3 (10)
multiple metastatic site	7 (47)	4 (31)	12 (39)
Liver metastases			
single	2 (13)	1 (8)	8 (26)
multiple	10 (67)	7 (54)	11 (35)
Previous adjuvant chemotherapy:	1 (6.5)	1 (8)	6 (19)
FA/5-FU bolus	-	-	3 (10)
Capecitabine	-	-	-
FOLFOX4	1 (6.5)	1 (8)	2 (6)
XelOx	-	-	1 (3)
Previous radiotherapy:	1 (6.5)	-	4 (13)
RT alone	-	-	-
RT+CT (5-FU c.i.)	-	-	2 (6)
RT+CT (XELOX)	1 (6.5)	-	2 (6)

c.i., continuous infusion; WHO, World Health Organization.

Pts' distribution according to extension of metastatic disease in c.35 G > A *KRAS *mutant, other *KRAS *mutant, and *KRAS *wild-type pts was, respectively: L-L 6 pts (40%), 7 pts (54%), and 12 pts (39%); O/MM 9 pts (60%), 6 pts (46%), 19 pts (61%).

### Activity and efficacy according to specific *KRAS *mutations

Activity and efficacy data in overall *KRAS *wild-type and mutant pts at a median follow-up of 21.5 months were previously reported [13]. Among 14 evaluable c.35 G > A *KRAS *mutant pts (Table 3), ORR was 71% (α 0.05, CI ± 26). We observed 10 objective responses: 9 partial responses (64%) and 1 complete response (CR) (7%) in a patient with single liver metastasis, who was progression-free at 60 months; 3 stable diseases (21%); 1 progressive disease (7%). Median PFS was 9 months (1+-60+ months): 10 events occurred and 5 pts (33%) were progression-free. Median OS was 14 months (1+-60+ months): 10 events occurred and 5 pts (33%) were alive. Liver metastasectomies were performed in 2 pts out of 15 (13%) and out of 6 pts with L-L metastases (33%); 1 R0 liver metastasectomy (17%). Clinical outcome according to extension of metastatic disease, L-L and O/MM [11,13], was: median PFS 9 and 7 months, median OS 11 and 14 months, respectively.

**Table 3:** Activity, efficacy and effectiveness of FIr-B/FOx regimen according to *KRAS *genotype

	c.35 G>A *KRAS *mutant		other *KRAS *mutant		*KRAS *wild-type	
	
	Intent-to-treat analysis		Intent-to-treat analysis		Intent-to-treat analysis	
	**Number**	**%**	**Number**	**%**	**Number**	**%**
	
Enrolled pts	15	100	13	100	31	100
Evaluable pts	14	93	13	100	30	97
Objective response	10	71 (CI ± 26)	8	61 (CI ± 26)	27	90 (CI ± 11)
partial response	9	64	8	61	23	76
complete response	1	7	-	-	4	13
Stable disease	3	21	2	15	2	7
Progressive disease	1	7	3	23	1	3
Median PFS, months	9		12		14	
range	1+-60+		3-37		1+-69+	
progression events	10	67	12	92	25	81
Median OS, months	14		39		38	
range	1+-60+		8-59+		1+-69+	
deaths	10	67	8	61.5	17	55
Liver metastasectomies	2		5		11	
number/overall pts	2/15	13	5/13	38	11/31	35
number/Pts with liver metastases	2/12	17	5/8	62.5	11/19	58
number/Pts with L-L metastases	2/6	33	5/7	71	10/12	83
Pathologic complete responses	-	-	2	40	-	-

L-L, liver-limited; OS, overall survival; PFS, progression-free survival; pts, patients.

Among 13 other than c.35 G > A *KRAS *mutant pts, ORR was 61% (α 0.05, CI ± 30). We observed 8 partial responses (61%), 2 stable diseases (15%), and 3 progressive diseases (23%). Median PFS was 12 months (3-37 months): 12 events occurred and 1 patient (8%) was progression-free >12 months. Median OS was 39 months (8-59+ months): 8 events occurred and 5 pts (38%) were alive. Liver metastasectomies were performed in 5 pts out of 13 (38%) and out of 7 (71%) with L-L metastases; 4 R0 liver resections (57%). Two pathologic CRs were obtained (15%) in pts with multiple L-L metastases, harboring codon 12 mutations, c.35 G > T and c.34 G > A: 1 patient progressed at 17 months, 1 patient was progression-free at 35 months. Clinical outcome according to extension of metastatic disease, L-L and O/MM [11,13], was: median PFS 16 and 12 months and median OS 44 and 21 months, respectively.

Activity and efficacy among 30 evaluable *KRAS *wild-type pts was previously reported [13]: ORR was 90% (α 0.05, CI ± 11). Four cCR were obtained (13%): 1 patient progressed at 22 months; 3 pts were progression-free at 69, 40 and 4 months. Median PFS was 14 months (1+-69+ months). Median OS was 38 months (1+-69+ months). Liver metastasectomies were performed in 11 pts: 35% of wild-type MCRC pts and 10 out of 12 L-L pts (83%). Among 18 *KRAS*/*BRAF *wild-type pts [13], ORR was 83% (α 0.05, CI ± 14). Median PFS was 13 months (4-44 months), median OS was 31 months (8-66+ months).

Among 44 evaluable other than c.35 G > A *KRAS *mutant plus *KRAS *wild-type pts, ORR was 81% (α 0.05, CI ± 12), median PFS was 13 months (1+-69+ months) and median OS was 34 months (1+-69+ months) (Table 4). Among 21 evaluable codon 12 *KRAS *mutant pts, ORR was 71% (α 0.05, CI ± 20), median PFS was 12 months (1+-60+ months) and median OS was 20 months (1+-60+ months). Among 7 c.35 G > T *KRAS *mutant pts, ORR was 57% (α 0.05, CI ± 40), median PFS was 12 months (3-5 months) and median OS was 21 months (11-46+ months). Among 4 codon 13 *KRAS *mutant pts, ORR was 75% (α 0.05, CI ± 49), median PFS was 12 months (7-37 months) and median OS was 44 months (8-59+ months). Among 3 c.38 G > A *KRAS *mutant pts, ORR was 67% (α 0.05, CI ± 65), median PFS was 12 months (7-37 months) and median OS was not reached (8-59+ months).

**Table 4:** Activity and efficacy according to *KRAS *genotype (intent-to-treat analysis)

	**ORR (%)**	PFS (m) range	OS (m) range
*KRAS *wild-type plus other than c.35 G>A *KRAS *mutant (44 pts)	81 (CI ± 12)	13	34
		1+-69+	1+-69+
*KRAS *wild-type (30 pts)	90 (CI ± 11)	14	38
		1+-69+	1+-69+
*KRAS *wild-type/*BRAF *wild-type (18 pts)	83 (CI ± 14)	13	31
		4-44	8-66+
other than c.35 G>A *KRAS *mutant (13 pts)	61 (CI ± 30)	12	39
		3-37	8-59+
c.35 G>A *KRAS *mutant (14 pts)	71 (CI ± 26)	9	14
		1+-60+	1+-60+
c.35 G>T *KRAS *mutant (7 pts)	57 (CI ± 40)	12	21
		3-25	11-46+

m, months; ORR, objective response rate; OS, overall survival; PFS, progression-free survival.

**Figure 1:**
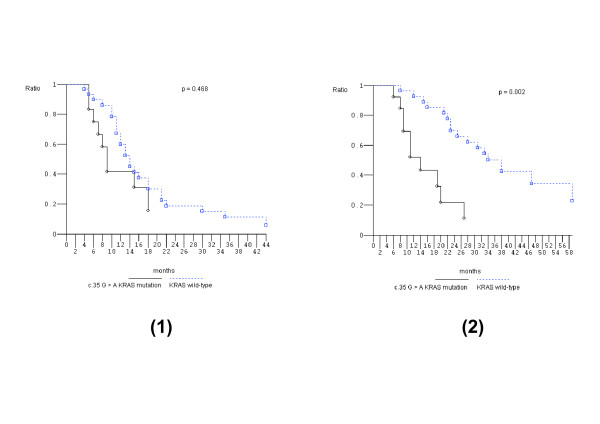
**Kaplan-Meier survival estimate**. c.35 G > A *KRAS *mutant patients versus *KRAS *wild-type patients. 1, progression-free survival; 2, overall survival.

**Figure 2:**
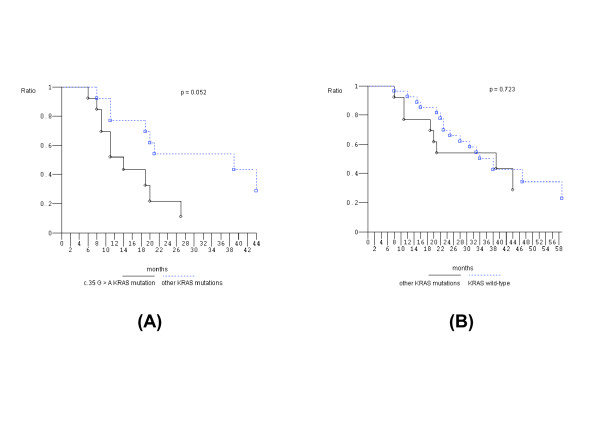
**Overall survival, Kaplan-Meier survival estimate**. **A**, c.35 G > A *KRAS *mutant patients versus other *KRAS *mutant patients; **B**, other *KRAS *mutant patients versus *KRAS *wild-type patients.

**Figure 3:**
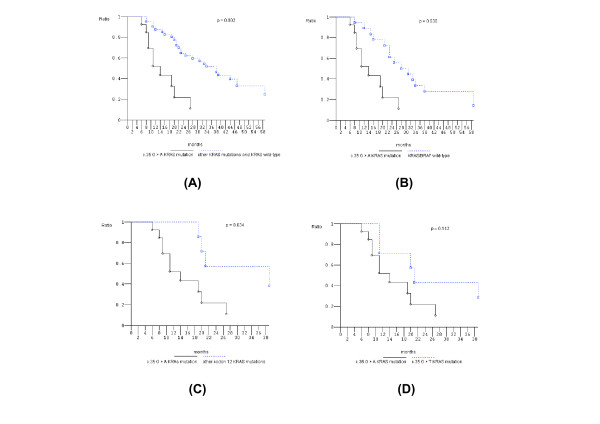
**Overall survival, Kaplan-Meier survival estimate**. **A**, c.35 G > A *KRAS *mutant patients versus other *KRAS *mutant plus *KRAS *wild-type patients; **B**, c.35 G > A *KRAS *mutant patients versus *KRAS/BRAF *wild-type patients; **C**, c.35 G > A *KRAS *mutant patients versus other codon 12 *KRAS *mutant patients; **D**, c.35 G > A *KRAS *mutant patients versus c.35 G > T *KRAS *mutant patients.

Figure 1 shows that PFS of c.35 G > A *KRAS *mutant pts compared to *KRAS *wild-type pts was not significantly different while OS was significantly worse (*P *= 0.002). In addition, c.35 G > A *KRAS *mutant pts compared to other than c.35 G > A *KRAS *mutant pts showed significantly worse OS (*P *= 0.05); other than c.35 G > A *KRAS *mutant pts compared to *KRAS *wild-type pts did not have different OS (Figure 2). *KRAS *c.35 G > A mutant pts also had significantly worse OS compared to: other than c.35 G > A *KRAS *mutant pts plus *KRAS *wild-type pts (*P *= 0.002); *KRAS/BRAF *wild-type pts (*P *= 0.03); and other codon 12 mutant pts (*P *= 0.03) (Figure 3). The prognostic relevance was not significantly different compared to c.35 G > T *KRAS *mutant pts (*P *= 0.142) (Figure 3).

## Discussion

The prognostic relevance of *KRAS *status, wild-type or mutant, is not significantly different in MCRC pts treated with BEV-containing chemotherapy. Reported median OS ranges from 29.9 to 38 months in *KRAS *wild-type and 19.9 to 21 months in *KRAS *mutant pts [4,5,8,13]. The addition of anti-EGFR or anti-VEGF molecules to doublet chemotherapy predicts a favorable clinical outcome in *KRAS *wild-type pts [2,5]. BEV addition to IFL compared to IFL significantly predicts prolonged PFS up to 9.3 months, but not increased OS and activity, in *KRAS *mutant pts [5,14]. BEV addition to triplet chemotherapy, according to FIr-B/FOx or FOLFOXIRI/BEV schedules, resulted in high activity and efficacy in *KRAS *wild-type and mutant MCRC pts [8,13]. In particular, *KRAS *mutant pts had an ORR of 67% and 71%, median PFS of 11 and 12.6 months, and median OS 20 months, respectively [8,13]. We recently reported a significantly favorable prognosis (PFS and OS) in *KRAS *wild-type L-L compared to O/MM pts [11,13]. Conversely, in *KRAS *mutant MCRC pts, median PFS and OS were not significantly affected by the extension of metastatic disease (L-L compared to O/MM) [11,13].

The prevalent c.35 G > A (G12D) *KRAS *mutation characterizes 10.3% of CRC and represents up to 30% of *KRAS *mutations [16]. In the present evaluation, 25.4% of MCRC pts harbored the c.35 G > A *KRAS *mutation and exhibited a high activity of the FIr-B/FOx intensive regimen (ORR 71%). Liver metastasectomies were performed in 13% of pts (33% of L-L disease), median PFS and OS were 9 and 14 months, respectively. In pts with the *KRAS *c.35 G > A mutation, activity and PFS were not significantly different, while OS was significantly worse compared to *KRAS *wild-type, *KRAS*/*BRAF *wild-type, and other codon 12 and 13 mutant pts. Median OS was not significantly different in other *KRAS *mutant compared to wild-type pts. This is the first report of a worse prognosis in *KRAS *c.35 G > A (G12D) mutant MCRC pts, treated with intensive triplet chemotherapy plus BEV.

Codon 12 *KRAS *mutations may increase aggressiveness by the differential regulation of KRAS downstream pathways associated with higher AKT/protein kinase B activation, bcl-2, E-catherin, β-catenin, and focal adhesion kinase overexpression, and RhoA underexpression, whereas codon 13 *KRAS *mutant cells show increased sensitivity associated with increased activation of the c-Jun-NH_2_-terminal kinase I pathway [19]. Several studies compared the prognostic roles of *KRAS *codon 12 with codon 13 mutations in CRC. RASCAL (Kirsten Ras in CRC) studies showed that the presence of the *KRAS *mutation significantly increased the risk of death by 26% [16,21]; the c.35 G > T (G12V) mutation, but not c.35 G > A (G12D) or c.35 G > C (G12A), represented an independent risk factor for recurrence and death and significantly increased the risk of death by 44% [21]. It also had a significantly worse impact on failure-free survival and OS, increasing the risk of recurrence or death by 30% [16], and up to 50% in Dukes' C cancers [16]. *KRAS *codon 12 mutations (in particular, c.35 G > T) were associated with inferior survival in patients with *KRAS*-wild-type/*BRAF*-wild-type cancers [29].

In MCRC pts, specific *BRAF *and *KRAS *mutations can confer different biological aggressiveness and effectiveness of treatment strategies; the balance between aggressiveness and effectiveness can differentiate prognosis, that is, median OS. Comparison of median OS in pts with different genotypes can discriminate this net prognostic effect. Thus, specific mutations and treatment strategies (medical regimens and secondary liver surgery, further lines of treatment) could be major parameters determining different prognoses in MCRC. The prevalent *BRAF *c.1799 T > A (V600E) mutation, characterizing 4.7% to 8.7% of CRC, demonstrated a negative prognostic effect compared to *BRAF *wild-type pts in MCRC pts treated with doublet chemotherapy alone or added to cetuximab, BEV and cetuximab plus BEV, with a median PFS of 5.6 to 8 months and median OS of 10.3 to 15.9 months [4,30,31]. The favorable predictive effect of cetuximab or BEV addition to chemotherapy was not significantly demonstrated in *BRAF *mutant MCRC pts [4,31,32]. Patients with tumors harbouring the *KRAS *c.35 G > T mutation and other mutations were associated with a worse outcome when receiving chemotherapy plus cetuximab, compared with chemotherapy alone [33].

In MCRC pts pre-treated with chemotherapy alone, the *KRAS *c.38 G > A mutation (G13D) confers a significantly worse prognosis [34]. Cetuximab or cetuximab plus chemotherapy significantly predicted increased OS (median 7.6 and 10.6 months, respectively) and PFS (median 4.0 and 4.1 months, respectively) compared to other *KRAS *mutations [34], and no different outcome was found compared to *KRAS *wild-type pts [34]. Recently, a retrospective pooled analysis confirmed the favorable predictive effect of c.38 G > A *KRAS *mutation in first line cetuximab-containing chemotherapy [33]: significantly improved PFS (median, 7.4 versus 6.0 months) and tumor response (40.5% versus 22.0) but not survival (median, 15.4 versus 14.7 months). Moreover, systematic reviews and meta analyses confirmed that *KRAS *c.38 G > A (G13D) mutant pts demonstrated a significantly favorable predictive effect of cetuximab-containing associations compared to other *KRAS *mutant MCRC, and significantly lower ORR, with no significantly different PFS and OS compared to *KRAS *wild-type pts [35,36]. In patients with MCRC treated with panitumumab or control therapy in first-or second-line chemorefractory settings, no consistent associations were found between tumors with specific *KRAS *mutations and patient outcome. Opposite findings were reported when panitumumab was combined with first line oxaliplatin, whereas similar data were reported when it was combined with second-line FOLFIRI [37].

Prospective studies should be developed to confirm the differential prognosis and predictive effect of chemotherapeutics and/or targeted agents in MCRC pts harboring *KRAS*/*BRAF *mutations, specifically *KRAS *c.35 G > A (G12D), c.35 G > T (G12V), c.38 G > A (G13D) mutations and *BRAF *c.1799 T > A (V600E).

## Conclusions

The prevalent *KRAS *c.35 G > A (G12D) mutant genotype has a significantly worse effect on the OS of MCRC pts treated with the first line FIr-B/FOx intensive regimen compared to wild-type pts or to pts harboring different other *KRAS *mutations, due to heterogeneous biological aggressiveness and the effectiveness of treatment strategies. The present findings should be verified in prospective trials of multidisciplinary strategies comparing clinical outcome in MCRC pts harboring specific mutations that differentially activate the downstream RAS-MAPK or PI3K pathways.

## Abbreviations

Anti-EGFR: anti-epidermal growth factor receptor; anti-VEGF: anti-vascular endothelial growth factor; BEV: bevacizumab; cCR: clinical complete responses; CR: complete response; CRC: colorectal cancer; CT: computed tomography; HR: hazard ratio; IFL: irinotecan, 5-fluorouracil, and leucovorin; L-L: liver-limited; MCRC: metastatic colorectal cancer; O/MM: other/multiple metastatic; ORR: objective response rate; OS: overall survival; PET: positron emission tomography; PFS: progression-free survival; pts: patients; RECIST: Response Evaluation Criteria in Solid Tumors.

## Competing interests

The authors declare that they have no competing interests.

## Authors' contributions

GB contributed to the conception and design of the study, in the provision of study materials of patients, in data analysis and interpretation and in the manuscript writing. ER contributed to the conception and design of the study, in data analysis and interpretation and in the manuscript writing. KC, TF, MT, EA participated in data analysis and interpretation. GB, KC, CF and ER provided clinical management and data on patients. DDG, AL, JCS provided molecular genetic analysis. All authors participated in the collection and/or assembly of data. All authors read and approved the final manuscript.

## Pre-publication history

The pre-publication history for this paper can be accessed here:

http://www.biomedcentral.com/1741-7015/11/59/prepub
